# Application of the Artificial Bee Colony Algorithm for Solving the Set Covering Problem

**DOI:** 10.1155/2014/189164

**Published:** 2014-04-16

**Authors:** Broderick Crawford, Ricardo Soto, Rodrigo Cuesta, Fernando Paredes

**Affiliations:** ^1^Pontificia Universidad Católica de Valparaíso, 2362807 Valparaíso, Chile; ^2^Universidad Finis Terrae, 7500000 Santiago, Chile; ^3^Universidad Autónoma de Chile, 7500000 Santiago, Chile; ^4^Escuela de Ingeniería Industrial, Universidad Diego Portales, 8370179 Santiago, Chile

## Abstract

The set covering problem is a formal model for many practical optimization problems. In the set covering problem the goal is to choose a subset of the columns of minimal cost that covers every row. Here, we present a novel application of the artificial bee colony algorithm to solve the non-unicost set covering problem. The artificial bee colony algorithm is a recent swarm metaheuristic technique based on the intelligent foraging behavior of honey bees. Experimental results show that our artificial bee colony algorithm is competitive in terms of solution quality with other recent metaheuristic approaches for the set covering problem.

## 1. Introduction

The set covering problem (SCP) is a classic problem in combinatorial analysis, computer science, and theory of computational complexity. It is a problem that has led to the development of fundamental technologies for the field of the approximation algorithms. Also it is one of the problems from the list of 21 Karp's NP-complete problems; its NP-completeness was demonstrated in 1972 [1].

SCP has many applications, including those involving routing, scheduling, stock cutting, electoral redistricting, and other important real life situations [2]. Although the best known application of the SCP is airline crew scheduling [3], where a given set of trips has to be covered by a minimum-cost set of pairings, a pairing being a sequence of trips that can be performed by a single crew.

Different solving methods have been proposed in the literature for the set covering problem. Exact algorithms are mostly based on branch-and-bound and branch-and-cut techniques [4–6], linear programing, and heuristic methods [7]. However, these algorithms are rather time consuming and can only solve instances of very limited size. For this reason, many research efforts have been focused on the development of heuristics to find a good result or near-optimal solutions within a reasonable period of time.

Classical greedy algorithms are very simple, fast, and easy to code in practice, but they rarely produce high quality solutions for their myopic and deterministic nature [8]. In [9] a greedy algorithm was improved incorporating randomness and memory into it and obtained promising results. Compared with classical greedy algorithms, heuristics based on Lagrangian relaxation with subgradient optimization are much more effective. The most efficient ones are those proposed by [10, 11].

Metaheuristics were also applied to the SCP as top-level general search strategies. An incomplete list of this kind of metaheuristics for the SCP includes genetic algorithms [12, 13], simulated annealing [14], tabu search [15], cultural algorithms [16, 17], and ant colony optimization [18]. For a deeper comprehension of effective algorithms for the SCP in the literature, we refer the interested reader to the survey by [7].

In this paper we propose a novel application of artificial bee colony (ABC) to solve SCP. This paper is organized as follows. In Section 2, we explain the problem. In Section 3, we describe the ABC framework. Our ABC proposal is described in Section 4. In Section 5, we present the experimental results obtained. Finally, in Section 6, we conclude the paper and give some perspectives for further research.

## 2. Problem Description

The set covering problem is a fundamental problem in the class of covering problems. Given a finite set *X* and a family *F* = *S*_1_, *S*_2_,…, *S*_*n*_ of subsets of *X* (i.e., *S*_*j*_⊆*X*, *j* = 1,…, *n*), the SCP aims to find a minimum cardinality *J*⊆{1,…, *n*} such that ⋃_*j*∈*J*_ *S*_*j*_ = *X*. The elements of *X* are called* points*. Given a *J*⊆{1,…, *n*}, a point is set to be covered if belongs to ⋃_*j*∈*J*_ *S*_*j*_. In the minimum-cost set covering problem each set *S*_*j*_, 1 ≤ *j* ≤ *n*, has a cost *c*_*j*_ and the problem is to find a *J*⊆{1,…, *n*}, where each point is covered and ∑_*j*∈*J*_ *c*_*j*_ is minimum. This minimum-cost optimization version of SCP is NP-hard.

Let us define the incidence matrix *A* of a set covering problem as follows. There are |*X*| rows in *A*, one for each point of *x*_*i*_ ∈ *X*, and *n* columns in *A*, one for each set *S*_*j*_. The entry *a*_*ij*_ at *A* (the entry at the intersection of the *i*th row and the *j*th column) is one if point *x*_*i*_ is in set *S*_*j*_; otherwise *a*_*ij*_ is zero. Table 1 shows an example of an incidence matrix.

For the upcoming reference cases, a general mathematical model of the SCP can be formulated as follows:

(1) Minimize  Z=∑j=1ncjxj

(2) Subject  to  ∑j=1naijxj≥1 ∀i={1,2,3,…,m}

(3)  xj∈{0,1} ∀j={1,2,3,…,n}.

Equation (1) is the objective function of the SCP, where *c*_*j*_ refers to the weight or cost of *j*-column and *x*_*j*_ is the decision variable. Equation (2) is a constraint to ensure that each row is covered by at least one column; *m* × *n* matrix *A* = (*a*_*ij*_) is a constraint coefficient matrix whose elements can be “1” or “0” to indicate the covering possibilities. Finally, (3) is the integrality constraint where the value *x*_*j*_ can be “1” if column *j* is activated (selected) or “0” otherwise.

## 3. Artificial Bee Colony Algorithm

ABC is one of the most recent algorithms in the domain of the collective intelligence. It was created by Dervis Karaboga in 2005, who was motivated by the intelligent behavior observed in the domestic bees to take the process of foraging [19].

ABC is an algorithm of combinatorial optimization based on populations, in which the solutions of the problem of optimization, the sources of food, are modified by the artificial bees that function as operators of variation. The aim of these bees is to discover the food sources with major nectar.

In the ABC algorithm, an artificial bee moves in a multidimensional search space choosing sources of nectar depending on its past experience and its companions of beehive or fitting its position. Some bees (exploratory) fly and choose food sources randomly without using experience. When they find a source of major nectar, they memorize their positions and forget the previous ones. Thus, ABC combines methods of local search and global search, trying to balance the process of the exploration and exploitation of the search space.

Although, the performance of different optimization algorithm is dependent on applications, some recent works demonstrate that the artificial bee colony is more rapid than either genetic algorithm or particle swarm optimization solving certain problems [20–24]. Additionally, ABC has demonstrated an ability to attack problems with a lot of variables (high-dimensional problems) [25].

### 3.1. Elements and Behavior

The model defines three principal components which are enunciated as follows.

*Food Source*. The value of a food source depends on many different factors, as its proximity to the beehive, wealth or the concentration of the energy, and the facility of extraction of this energy. 

*Employed Bees or Workers*. They are associated with a current food source, or in exploitation, they take with them information about this source, especially the distance, location, and profitability, to share this with a certain probability with other companions. 

*Unemployed or Exploratory Bees*. They are in constant search of a food source. There are two types:

- scouts: they are the ones in charge of searching in the environment that surrounds the beehive for new sources of food.
- onlookers (curious or in wait): they look for a food source across the information shared by the employees or by other explorers in the nest.

### 3.2. Biological Behavior

The exchange of information between the bees is the most important incident in the formation of a collective knowledge, since the meaning of this interaction the bees will decide the behavior that must take the beehive. The principal ways of bee behaviour are

- the incorporation to a source of nectar: the communication between the bees related to the quality of food sources is realized in the zone of dance (dance of the bees), where with the information obtained about all the sources of food that are available, they decide which of all the sources is the most profitable to join.
- the abandon of a source: by means of the dance it is determined if a source is no longer profitable and consequently it must be abandoned.

### 3.3. Artificial Behavior

In Table 2 the elements of the ABC are described in a general way.

The pseudocode of artificial bee colony is as in Algorithm 1.

The procedure for determining a food source in the neighborhood of a particular food source which depends on the nature of the problem. Karaboga [26] developed the first ABC algorithm for continuous optimization. The method for determining a food source in the neighborhood of a particular food source is based on changing the value of one randomly chosen solution variable while keeping other variables unchanged. This is done by adding to the current value of the chosen variable the product of a uniform variable in [−1, 1] and the difference in values of this variable—current food source—and some other randomly chosen food source. This approach cannot be used for discrete optimization problems for which it generates at best a random effect.

Singh [27] subsequently proposed a method, which is appropriate for subset selection problems. In his model, to generate a neighboring solution, an object is randomly dropped from the solution and in its place another object, which is not already present in the solution, is added. The object to be added is selected from another randomly chosen solution. If there are more than one candidate object for addition, then ties are broken arbitrarily.

This approach is based on the idea that if an object is present in one good solution, then it is highly likely that this object is present in many good solutions. This method provides another advantage, consisting in that if the method fails to find an object different from the others objects in the original solution, this means that the two solutions are equal, such situation is called “collision” and it is resolved by making the employed bee associated with the original solution, a scout bee eliminating duplication.

## 4. Description of the Proposed Approach to Solve SCP

**Step 1 (initialization)**

This step includes initializing the parameters of ABC as size of the colony, number of workers and curious (onlookers or “in wait”) bees, limit of attempts, and maximum number of cycles.

**Step 2 (generation of initial population)**

**Figure 1:**
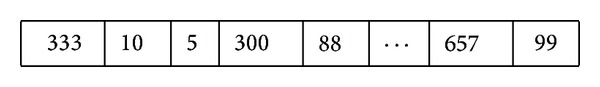
Representation of a solution.

To generate the initial population by every row—SCP constraint—a column—SCP variable—is selected at random from the set of columns with covering possibilities. A solution is represented by means of an entire vector as shown in Figure 1 keeping the columns considered in the solution. Then, we use an integer encoding as the encoding rule.

**Step 3 (evaluation of the fitness of the population)**

The fitness function is equal to the objective function of the SCP (1).

**Step 4 (modification of position and selection of sites for worker bees)**

A hard-working bee modifies its position by means of the creation of a new solution based on a different food source selected randomly. It sees if at least it has a different column, in case of having not even a different column, the hard-working bee is transformed to an explorer in order to eliminate duplicated solutions. In opposite case, it proceeds to add a certain random number of columns between 0 and the maximum number of columns to be added.

After this, it proceeds to eliminate a certain random number of columns between 0 and the maximum number of columns to be eliminated. In case that new solution does not meet constraints, it is repaired. The fitness of the solution is evaluated; if the fitness (cost) is minor than the solution obtained in the beginning, the solution is replaced. In opposite case, it increases the number of attempts for improving this solution (limit).

**Step 5 (recruiting curious bees for the selected sites)**

A curious bee evaluates the information of the nectar through the workers and it chooses a source of food with the fitness proportionate selection method or roulette-wheel selection.

**Step 6 (modification of position for the curious bees)**

They work alike to hard-working bees in Step 4.

**Step 7 (leaving a source exploited by the bees)**

If the solution representing a source of food does not improve for a predetermined number of attempts (limit), then the source of food is left and is replaced by a new source of food generated as in Step 1.

**Step 8**

This step involves memorizing the best solution and increasing the counter of the cycle.

**Step 9**

The process stops if the criteria of satisfaction expire; in opposite case return to Step 3.

## 5. Experimental Results

The ABC algorithm has been implemented in C in a 2.5 GHz Dual Core with 4 GB RAM computer, running windows 7.

Parameter values have a profound influence on the performance of ABC. The parameters were empirically adjusted, we determined their values in an experimental way, and for each parameter, a set of candidate values were considered. We modified the value of one parameter while keeping the others fixed. According to the best results, as parameter values in our experiments, we use ABC runs 1000 iterations with a population of 200 bees, where 100 corresponds to hard-working and 100 to curious. Limit = 50, maximum number of columns to add = 0.5% of columns in the SCP instance, and maximum number of columns to eliminate = 1.2% of the SCP instance.

These parameters showed good results, but they cannot be the ideal ones for all the instances. ABC has been tested on 65 standard non-unicost SCP instances available from OR Library at http://people.brunel.ac.uk/~mastjjb/jeb/info.html.  Table 3 summarizes the characteristics of each of these sets of instances, each set contains 5 or 10 problems and the column labeled Density shows the percentage of nonzero entries in the matrix of each instance. ABC was executed 30 times on each instance, each trial with a different random seed.

### 5.1. Comparison with Other Works

In comparison with very recent works solving SCP—with cultural algorithms [16] and ant colony + constraint programming techniques [28]—our proposal performs better than the SCP instances reported in those works.

In order to bring out the efficiency of our proposal, the solutions of the complete set of instances have been compared with other metaheuristics. We compared our algorithm solving the complete set of 65 standard non-unicost SCP instances from OR Library with the newest ACO-based algorithm for SCP in the literature: ant-cover + local search (ANT + LS) [29], genetic algorithm (GA) proposed by Beasley and Chu (1996) [13], and simulated annealing (SA) proposed by Brusco et al. (1999) [14].

Tables 4 and 5 show the detailed results obtained by four algorithms. Column 2 reports the optimal or the best known solution value of each instance. The third and fourth columns show the best value and the average obtained by our ABC algorithm in the 30 runs (trials). The next columns show the average values obtained by GA, SA, and ANT + LS, respectively. The last column shows the relative percentage deviation (RPD) value over the instances tested with ABC. The quality of solutions can be evaluated using the RPD; its value quantifies the deviation of the objective value *Z* from *Z*_opt_ which in our case is the best known cost value for each instance. This measure is computed as follows:

(4) $$\begin{array}{c} \text{RPD}=\frac{\left(Z-Z_{\text{opt}}\right)}{Z_{\text{opt}}}\times 100. \end{array}$$

Examining Tables 4 and 5, we observe the following.

- ABC is able to find the optimal solution consistently, that is, in every trial, for 43 of 65 problems.
- ABC is able to find the best known value in all instances of Table 5.
- ABC is able to find the best known value in all trials of Table 5.
- ABC has higher success rate compared to genetic algorithm, simulated annealing, and ants in sets NRE, NRF, NRG, and NRH. The RPD of BEE is 0.00%, the RPD of GA is 1.04%, the RPD of SA is 0.72%, and the RPD of ANT + LS is 0.86%.
- ABC can obtain optimal solutions in some instances where the other metaheuristics failed.

### 5.2. Convergence to the Best Solution

Our approach shows an excellent tradeoff between the quality of the solutions obtained and the computational effort required. In all cases, ABC converged very quickly (mainly from the 10th iteration) and its computation time in the runs was less than 2 seconds (except for NRG and NRH instances where the computation time was less than 30 secs).

**Figure 2:**
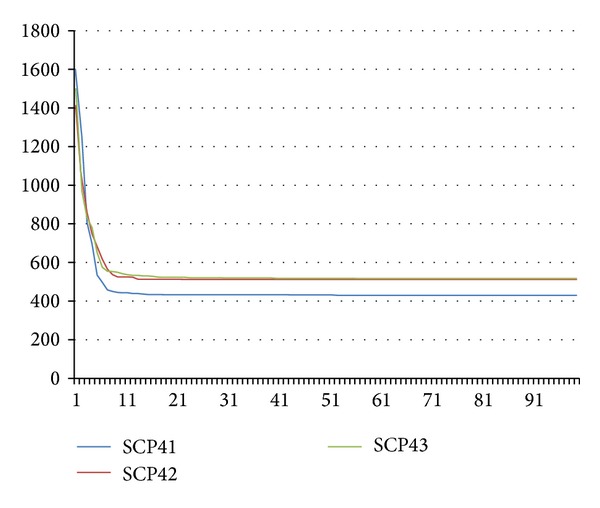
Convergence analysis to a better solution (benchmarks: SCP41, SCP42, and SCP43).

**Figure 3:**
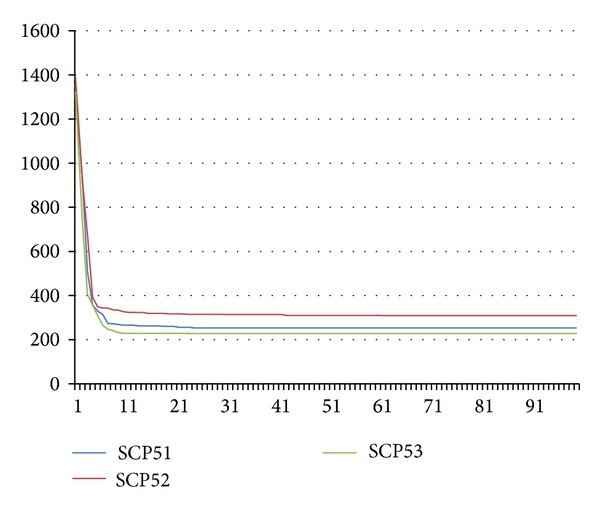
Convergence analysis to a better solution (benchmarks: SCP51, SCP52, and SCP53).

Figures 2 and 3 illustrate how ABC converges through the iterations to a better solution. We consider only 3 problems per chart in favor of clarity and readability: scp41, scp42, and scp43 for the first chart and scp51, scp52, and spc53 for the second one. *x*-axis represents the iteration number while *y*-axis represents the reached fitness value.

## 6. Conclusion

In this paper we have presented an ABC algorithm for the SCP. We have performed experiments throught several ORLIB instances; our approach has been shown to be very effective, providing an unattended solving method, for quickly producing solutions of a good quality. Experiments showed interesting results in terms of robustness, where using the same parameters for different instances gave good results.

The promising results of the experiments open up opportunities for further research. We visualize different directions for future work as follows.

- The fact that the presented algorithm is easy to implement clearly implies that ABC could also be effectively applied to other combinatorial optimization problems.
- An interesting proposal by Teodor Crainic et al. at [30] involves parallelizing strategies for metaheuristics. The author sets a basis on the idea that the central goal of parallel computing is to speed up computation by dividing the work load among several threads of simultaneous execution; then a type of metaheuristic parallelism could come from the decomposition of the decision variables into disjoint subsets. The particular heuristic is applied to each subset and the variables outside the subset are considered fixed.
- An interesting extension of this work would be related to hybridization with other metaheuristics or applying a hyperheuristic approach [31].
- The use of autonomous search (AS) represents a new research field, and it provides practitioners with systems that are able to autonomously self-tune their performance while effectively solving problems. Its major strength and originality consist in the fact that problem solvers can now perform self-improvement operations based on analysis of the performances of the solving process [32–34].
- Furthermore, we are considering to use different preprocessing steps from the OR literature, which allow to reduce the problem size [35].

## Figures and Tables

**Algorithm 1:**
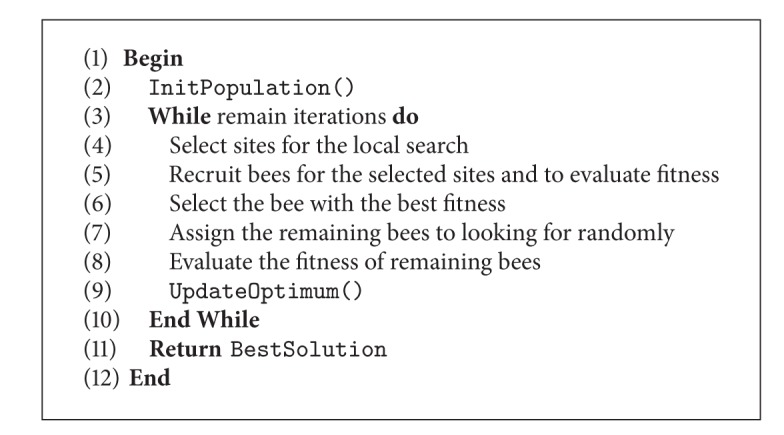
ABC pseudocode.

**Table 1:** Incidence matrix example.

$A=\;\;\begin{array}{cccccc} & S_{1} & S_{2} & S_{3} & S_{4} & S_{5}\\ x_{1} & 0 & 0 & 1 & 0 & 1\\ x_{2} & 0 & 1 & 0 & 0 & 0\\ x_{3} & 1 & 0 & 1 & 0 & 0\\ x_{4} & 0 & 1 & 0 & 0 & 1\\ x_{5} & 0 & 0 & 0 & 0 & 1\\ x_{6} & 0 & 0 & 0 & 1 & 0\\ x_{7} & 1 & 0 & 1 & 1 & 0\\ x_{8} & 0 & 1 & 1 & 0 & 0\\ x_{9} & 1 & 0 & 0 & 0 & 1\\ x_{10} & 0 & 1 & 0 & 0 & 0 \end{array}$	

**Table 2:** Summary of ABC main elements.

Generation of food sources	A solution to the optimization problem is a food source. It moves in a random way and with base in the low and superior limits of every variable of the problem

Working bees	Their number is proportional to the number of food sources, where for every source there is a working bee, and its function is to evaluate and to modify the current solutions to improve them (it looks for new sources near the current one). If the new position is not better than the current, it will keep the original position

Bees in wait	The number of bees in wait must be proportional to the number of sources. These bees will choose a food source, based on the information of the working bees by means of the waggle dance, where the food source with better value of objective function is selected

Scout bees	These bees generate new sources of food in a random way to replace existing sources that have not been improved

Limit	It defines the maximum number of cycles that a food source can keep without improving before being replaced. The limit increases from the source that is not modified by the bees; already they are used or in wait, up to obtaining its maximum allowed value. After this, the scout bees take charge of initializing the limit to 0 for every new generated position. The limit is initialized to 0 whenever a source is modified (improved) by a used bee or in wait

Column adding	When solving SCP, it defines the number of columns to be added to the current food source

Column elimination	When solving SCP, it defines the number of columns to be eliminated from the current food source

**Table 3:** Details of the 65 test instances.

Instance set	Number of instances	*m*	*n*	Cost range	Density (%)	Optimal solution
4	10	200	1000	[1,100]	2	Known
5	10	200	2000	[1,100]	2	Known
6	5	200	1000	[1,100]	5	Known
A	5	300	3000	[1,100]	2	Known
B	5	300	3000	[1,100]	5	Known
C	5	400	4000	[1,100]	2	Known
D	5	400	4000	[1,100]	5	Known
NRE	5	500	5000	[1,100]	10	Unknown
NRF	5	500	5000	[1,100]	20	Unknown
NRG	5	1000	10000	[1,100]	2	Unknown
NRH	5	1000	10000	[1,100]	5	Unknown

**Table 4:** Experimental results—instances with optimal.

Instance	Optimum	Best value found	ABC Avg.	GA Avg.	SA Avg.	ANT-LS Avg.	RPD (%)
4.1	429	430	430.5	429.7	—	429	0.35
4.2	512	512	512	512	—	512	0
4.3	516	516	516	516	—	516	0
4.4	494	494	494	494.8	—	494	0
4.5	512	512	512	512	—	512	0
4.6	560	561	561.7	560	—	560	0.30
4.7	430	430	430	430.2	—	430	0
4.8	492	493	494	492.1	—	492	0.41
4.9	641	643	645.5	643.1	—	641	0.70
4.10	514	514	514	514	—	514	0
5.1	253	254	255	253	—	253	0.79
5.2	302	309	310.2	303.5	—	302	2.72
5.3	226	228	228.5	228	—	226	1.11
5.4	242	242	242	242.1	—	242	0
5.5	211	211	211	211	—	211	0
5.6	213	213	213	213	—	213	0
5.7	293	296	296	293	—	293	1.02
5.8	288	288	288	288.8	—	288	0
5.9	279	280	280	279	—	279	0.36
5.10	265	266	267	265	—	265	0.75
6.1	138	140	140.5	138	—	138	1.81
6.2	146	146	146	146.2	—	146	0
6.3	145	145	145	145	—	145	0
6.4	131	131	131	131	—	131	0
6.5	161	161	161	161.3	—	161	0
A.1	253	254	254	253.2	—	253	0.40
A.2	252	254	254	253	—	252	0.79
A.3	232	234	234	232.5	—	232.8	0.86
A.4	234	234	234	234	—	234	1.10
A.5	236	237	238.6	236	—	236	0
B.1	69	69	69	69	—	69	0
B.2	76	76	76	76	—	76	0
B.3	80	80	80	80	—	80	0
B.4	79	79	79	79	—	79	0
B.5	72	72	72	72	—	72	0
C.1	227	230	231	227.2	—	227	1.76
C.2	219	219	219	220	—	219	0
C.3	243	244	244.5	246.4	—	243	0.62
C.4	219	220	224	219.1	—	219	2.28
C.5	215	215	215	215.1	—	215	0
D.1	60	60	60	60	—	60	0
D.2	66	67	67	66	—	66	1.52
D.3	72	73	73	72.2	—	72	1.39
D.4	62	63	63	62	—	62	1.61
D.5	61	62	62	61	—	61	1.64

**Table 5:** Experimental results—instances with best known solution.

Instance	Optimum	Best value found	ABC Avg.	GA Avg.	SA Avg.	ANT-LS Avg.	RPD (%)
NRE.1	29	29	29	29	29	29	0
NRE.2	30	30	30	30.6	30	30	0
NRE.3	27	27	27	27.7	27	27	0
NRE.4	28	28	28	28	28	28	0
NRE.5	28	28	28	28	28	28	0
NRF.1	14	14	14	14	14	14	0
NRF.2	15	15	15	15	15	15	0
NRF.3	14	14	14	14	14	14	0
NRF.4	14	14	14	14	14	14	0
NRF.5	13	13	13	13.7	13.7	13.5	0
NRG.1	176	176	176	177.7	176.6	176	0
NRG.2	154	154	154	156.3	155.3	155.1	0
NRG.3	166	166	166	167.9	167.6	167.3	0
NRG.4	168	168	168	170.3	170.7	168.9	0
NRG.5	168	168	168	169.4	168.4	168.1	0
NRH.1	63	63	63	64	64	64	0
NRH.2	63	63	63	64	63.7	67.9	0
NRH.3	59	59	59	59.1	59.4	59.4	0
NRH.4	58	58	58	58.9	58.9	58.7	0
NRH.5	55	55	55	55.1	55	55	0
